# Long-Term High Flow Nasal Cannula Therapy in Primary and Secondary Bronchiectasis

**DOI:** 10.3390/healthcare11091250

**Published:** 2023-04-27

**Authors:** Francesca Simioli, Giuseppe Fiorentino, Rosa Cauteruccio, Antonietta Coppola, Pasquale Imitazione, Antonella Marotta, Valentina Di Spirito, Anna Annunziata

**Affiliations:** Department of Respiratory Pathophysiology and Rehabilitation, Monaldi Hospital, A.O. deiColli, 80131 Naples, Italy

**Keywords:** rehabilitation, cystic fibrosis, heated and humidified therapy, mucus, COPD

## Abstract

Background: Bronchiectasis is the consequence of chronic bronchial inflammation, inappropriate mucus clearance, bacterial colonization, and recurrent or chronic infection. High flow therapy (HFT) is a type of non-invasive respiratory therapy, usually delivered through a nasal cannula interface (HFNC). It delivers heated and humidified air with a stable fraction of inspired oxygen and a wide range of possible flow rates. Aim of the study: Determine the effectiveness of HFNC as add-on therapy in adult primary and secondary bronchiectasis with frequent acute exacerbations (AEs) and/or hospitalizations. Methods: This is a single-center crossover study on long-term home therapy with HFNC in adult bronchiectasis. Pharmacological therapy included pulse therapy with mucolytics and bronchodilators. After one year, all patients were switched to additional HFNC. The temperature range was 31–37 °C. The flow range was 35–60 L/m. FiO_2_ was 0.21. Results: Seventy-eight patients completed the follow-up; 54% were females; the median age was 70 years (IQR 60–76). The etiology of bronchiectasis was mainly post-infective (51%), COPD related (26%), and congenital (11%). AEs at baseline were 2.81 (±2.15). A significant reduction in AEs was observed after 24 months with a mean of 0.45 (±0.66) (f-ratio value 79.703. *p*-value < 0.00001). No significant difference was observed after HFNC therapy on FEV_1_ (2.39 ± 0.87 vs. 2.55 ± 0.82; f-ratio 0.79. *p*-value 0.45) and FVC (2.73 ± 0.88 vs. 2.84 ± 0.90; f-ratio 0.411. *p*-value 0.66). A significant reduction in mMRC score was observed after HFNC therapy (2.40 ± 0.81 vs. 0.97 ± 0.97 at 2 months vs. 0.60 ± 0.78 at 24 months; f-ratio value 95.512. *p*-value < 0.00001). Conclusions: HFNC is a well-tolerated add-on therapy for adult bronchiectasis. Dyspnea improved after 2 months and further after 2 years. The exacerbation rate decreased during the 2 years follow-up. No significant difference was observed in lung function.

## 1. Background

Bronchiectasis is defined as the pathological and irreversible dilatation of the small and medium-sized bronchi [1]. Bronchiectasis is not a disease in its own right but rather a consequence of chronic bronchial inflammation, inappropriate mucus clearance, bacterial colonization, and recurrent or chronic infection. It is characterized by thickening of the bronchial wall and increased sputum production and chronic cough. A variety of systemic and respiratory diseases may be complicated by bronchiectasis. In Europe, it is often associated with patients affected by cystic fibrosis [1]. However, in many cases, no causes are found. Lung mucus retention is a characteristic of bronchiectasis, predisposing to recurrent respiratory infection [2]. The sensation of retained mucus and a concomitant high frequency of coughing and sputum production can furthermore be distressing to the patient. The importance of mucociliary clearance as a first-line defense mechanism of the bronchial tree is well established [3].

Humidified and heated high flow therapy (HFT) is a type of non-invasive respiratory therapy, usually delivered through a nasal cannula interface (HFNC). It delivers heated and humidified air with a stable fraction of inspired oxygen (FiO_2_) at flow rates up to 60 L/min [4]. HFT has several physiologic effects, such as humidification and improved mucociliary clearance, washout of dead space, and alveolar recruitment, representing a valid therapy for various respiratory diseases. HFT is also used in the acute setting, representing an emerging ventilatory support [5].

Humidification therapy is a promising approach in patients affected by bronchiectasis non-related to cystic fibrosis (CF) because HFT improves mucociliary clearance [6] and this is important for breaking the “vicious cycle” of recurrent infections and airway inflammation [7]. Good et al. found that HFT significantly decreased exacerbation days, increased time to exacerbation, and reduced exacerbation frequency compared to usual care in forty-five bronchiectasis patients [8]. In a recent prospective observational study, Crimi et al. describe the effect of HFT in the acute setting. Fifteen cases of acute exacerbation of chronic obstructive pulmonary disease (AECOPD) and bronchiectasis showed improvement in dyspnea, gas exchange, and mucus clearance with HFNC [9].

The major burden of bronchiectasis is related to exacerbations, loss of working days, and hospitalizations. Those outcomes require an adequate amount of time to be assessed with reliability. For this reason, in our study, we selected patients with documented bronchiectasis and also affected by a severe clinical status and set the follow-up at 24 months from HFNC initiation.

## 2. Aim of the Study

The aim of the study was to test long-term HFT for all-causes adult bronchiectasis with frequent exacerbations and/or hospitalizations. All patients underwent pharmacological therapy and a rehabilitation program. After a 12-month run-in period, additional HFT was started.

## 3. Methods

**Figure 1:**
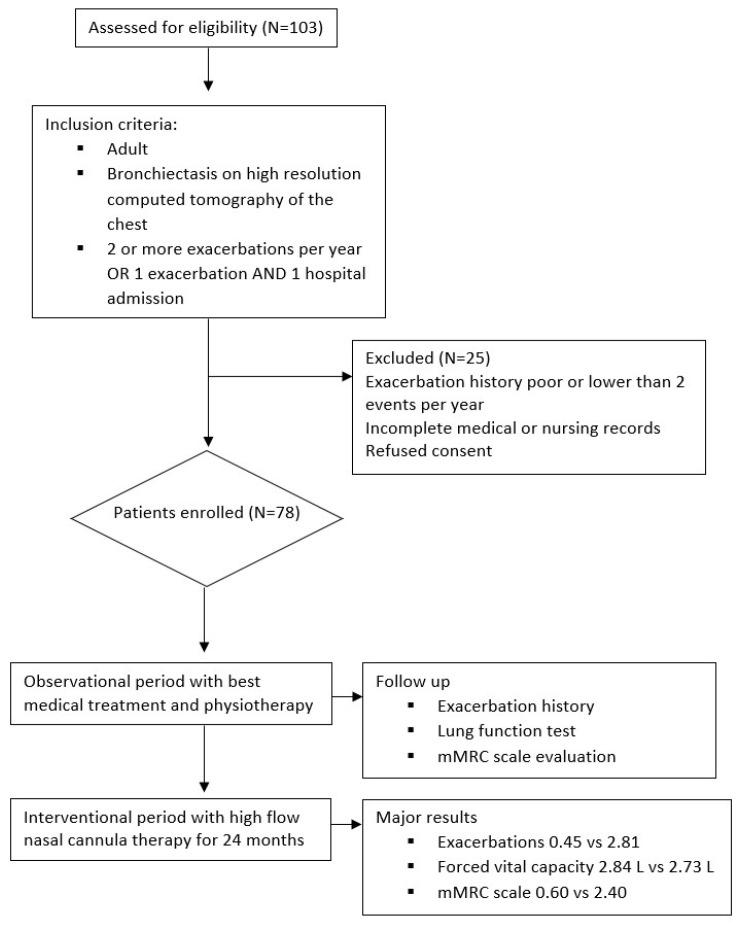
Flowchart of the study.

This is a single-center crossover study on long-term home therapy with HFNC in adult bronchiectasis. One hundred three bronchiectasis patients were visited at our Respiratory Rehabilitation outward clinic from September 2019 to June 2020. Bronchiectasis was diagnosed based on a high-resolution computed tomography (HRCT) of the chest performed within twelve months before enrollment. A total of 87 patients showed a severe clinical syndrome characterized by 2 or more exacerbations in the last year and/or at least 1 hospital admission caused by bronchiectasis. Medical history was recorded at baseline, including concomitant diseases, previous respiratory tract infections, antibiotics use, hospital admissions, and concomitant pharmacological therapy. The inclusion criteria were: age ≥ 18 years, radiological evidence of bronchiectasis on HRCT, at least 2 exacerbations and/or hospitalizations for respiratory causes, smoke cessation > 3 months, and ability to use electronic devices. The exclusion criteria were: clinically important pulmonary diseases other than bronchiectasis, respiratory exacerbation in the last 7 days, partial oxygen pressure (pO2) < 60 mmHg on arterial blood gas, long-term exertional oxygen therapy (LTOT) > 2 Liters per minute, chronic use of any non-invasive positive pressure ventilation device, active smoking or vaping of any products within the 3 months prior to enrollment, ongoing SARS-CoV-2 infection, significant COVID-19 illness within the 6 months prior to enrolment, active tuberculosis, recent positive TB test, unstable heart, kidney, or liver diseases at the enrollment time, unstable cardiovascular disorder, heart failure NYHA functional class III–IV, left ventricular ejection fraction < 30%, cor pulmonale, pulmonary arterial hypertension and/or right ventricular failure, scheduled major surgical procedure during the course of the study, and history of alcohol or drug abuse within the past year. A total of 103 patients were assessed for eligibility, and 25 patients were excluded. Figure 1 reports the flowchart. All severe bronchiectasis were enrolled in a program of 4 weeks of respiratory rehabilitation. Pharmacological therapy included pulse therapy with mucolytics, and N-acetylcysteine (NAC) was prescribed once daily for 15 consecutive days per month to be repeated every month. The chronic endobronchial inflammation/infection and profuse airway secretions which dominate pulmonary diseases such as CF make the use of mucolytic agents an appealing treatment for bronchiectasis. Mucolytic agents given as part of chronic maintenance treatment could conceptually be hoped to be effective in reducing acute exacerbations; however, until the last decades, there was little evidence of this benefit, and NAC found no benefit on lung function in short-term trials. Bronchodilators were prescribed for bronchial obstruction based on GOLD guidelines for COPD [10]. After one year, all patients were switched to additional HFNC. The temperature was initially set at 37 °C and eventually reduced based on the patient’s tolerance. The flow was initially set at 45 L/min and eventually modified based on personal comfort. A total of 78 patients agreed to continue HFNC at home and were included in this study. All subjects completed the 2-year follow-up. 

Respiratory lung function was tested by spirometry at baseline and after HFNC initiation, at 2 months and at 24 months. Absolute values of forced expiratory volume in 1 s (FEV_1_) and forced vital capacity (FVC) were measured in liters (L). Dyspnea was measured by the Modified British Medical Research Council Questionnaire (mMRC) at baseline, after HFNC initiation at 2 months and at 24 months. The occurrence of respiratory exacerbations and hospitalizations were monitored via monthly phone calls and via physical examination every 6 months up to 24 months after HFNC initiation. 

The study was approved by the local ethics committee of the University of Campania “Luigi Vanvitelli” and A.O. dei Colli in accordance with the 1976 Declaration of Helsinki and its later amendments. All subjects consented to participate.

Acute exacerbations (AEs) per year are the primary outcome. An AE is defined as a sustained worsening of the patient’s condition from the stable state and beyond normal day-to-day variations that is acute in onset and is clinically characterized by 2 symptoms among increased dyspnea, sputum purulence, and sputum volume. An AE may warrant additional treatment (e.g., antibiotic, systemic corticosteroid, bronchodilators) [11]. Secondary outcomes are hospital admissions, lung function measured with FEV_1_ and FVC, dyspnea, and functional limitation scored by the mMRC scale. The mMRC scale stratifies the severity of dyspnea in respiratory diseases. The baseline functional disability due to dyspnea is scored in five classes (from 0 to 4), corresponding to an increasing level of perceived symptoms while approaching common daily activities. This explains why the scale strongly predicts autonomy and occupational performance and reflects the impact of the disease on the patient’s quality of life. In daily practice, dyspnea level is usually measured by the mMRC scale. It is very easy to understand and quite rapid to perform for the majority of patients. The mMRC has a prognostic value and was, thus, included in all simplified prognostic scores, such as the Body mass index–airflow Obstruction–Dyspnea, and Exercise (BODE) index. Moreover, evaluation of the level of dyspnea by the mMRC is now used to categorize COPD symptomatic burden in the last GOLD recommendations and provides useful information about disease-induced disability. However, its unidimensional structure and limited number of degrees are well-recognized limitations. Dyspnea is among the predominant symptoms in bronchiectasis, both in stable condition and during exacerbations, and appears now as a major index of disease severity and a prominent target of treatment. Dyspnea has been shown to be weakly associated with the most common lung function parameters, particularly with FEV_1_, suggesting the contribution of many other factors. Comorbidities, defined as other specific chronic diseases, are frequently associated with COPD, and their importance is being increasingly recognized. They impact many aspects of the disease and interfere with its natural history. For example, high rates of cardiovascular diseases (e.g., chronic heart failure) and mood disorders (e.g., anxiety and depression) have been reported in COPD patients and suggested as contributing to dyspnea [12].

The results of categorical variables are reported as numbers and percentages; continuous variables are reported as median and interquartile range (IQR) or mean and standard deviation (SD). The dependent variables were compared with the one-way ANOVA test for the overall difference. Tukey’s HSD (honestly significant difference) was used to assess pairwise comparison. A *p*-value < 0.05 was considered statistically significant. A multivariate regression analysis was performed to describe how AEs change with varying HFNC settings, such as flow and temperature, as well as hours per day of utilization. A total of 78 adult bronchiectasis patients were enrolled and completed the follow-up; 42 subjects (54%) were females. The median age was 70 years (interquartile range [IQR] 60–76). The etiology of bronchiectasis among our population was mostly post-infective (51%) and COPD related (26%). Congenital bronchiectasis (11%) includes cystic fibrosis (CF) and primary ciliary dyskinesia (PCD). The baseline features are described in Table 1.

During HFNC therapy, the mean tolerated temperature was 34 °C. The flow was set between 35 and 60 L/min, with a mean of 45 L/min. No additional oxygen was needed. 

## 4. Results

**Figure 2:**
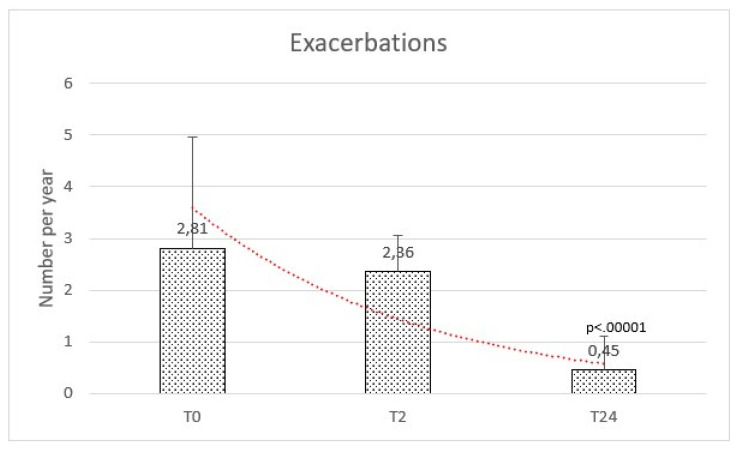
Exacerbations were comparable at baseline (T0) and after 2 months (T2), while reduced after the high flow therapy at 2 years (T24).

The rate of exacerbations per year at baseline ranged between 1 and 9, with a mean of 2.81 (±2.15). At 2 months after HFNC initiation, the mean was 2.36 (±0.69), whereas a significant reduction of exacerbations was observed after 24 months with a mean of 0.45 (±0.66) [f-ratio value 79.703. *p*-value < 0.00001. T1:T2 Q = 0.25 (*p* = 0.98). T1:T3 Q = 15.34 (*p* = 0.000001). T2:T3 Q = 15.59] (Figure 2). 

**Figure 3:**
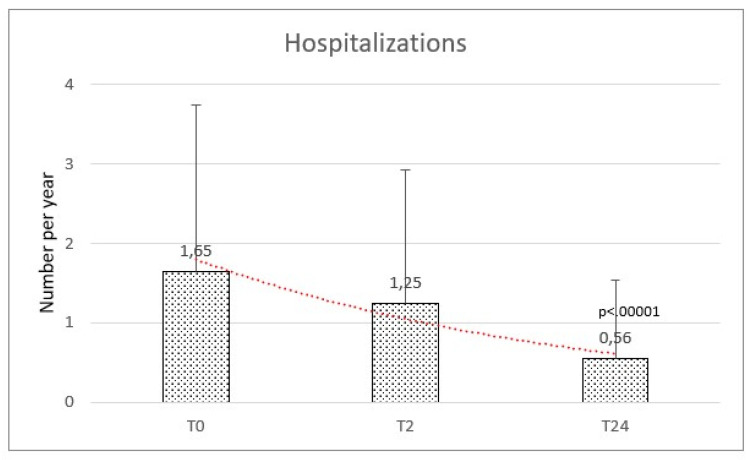
Hospitalizations were comparable at baseline (T0) and after 2 months (T2), while reduced after the high flow therapy at 2 years (T24).

Hospital admission in the year before HFNC initiation was frequent, with 45 records among this population at our clinic. The rate of hospitalization per year at baseline was 1.65 (±2.10) and significantly reduced at the final follow-up with a mean of 0.56 (±0.98) (f-ratio value 76.165. *p*-value < 0.00001) (Figure 3).

**Figure 4:**
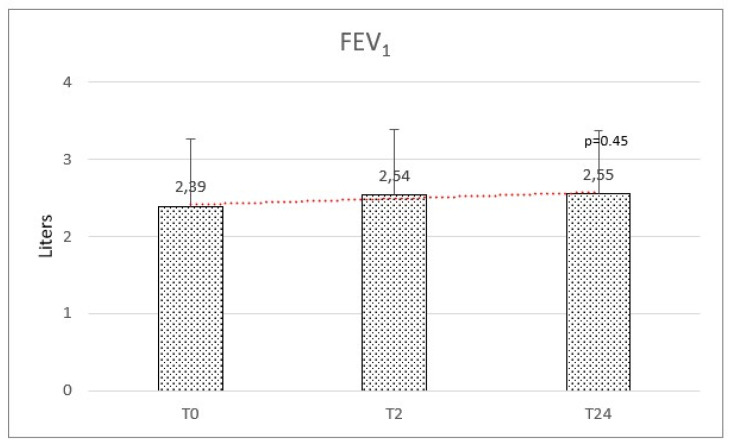
Forced expiratory volume in the first second (FEV1) at baseline (T0), after 2 months (T2), and after 2 years (T24) was not significantly modified.

**Figure 5:**
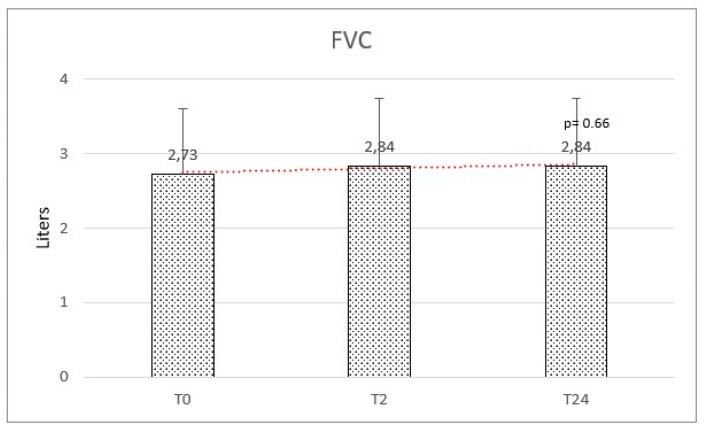
Forced vital capacity (FVC) at baseline (T0), after 2 months (T2), and after 2 years (T24) was not significantly modified.

Respiratory function at baseline was: mean FEV_1_ of 2.39 (±0.87) and a mean FVC of 2.73 (±0.88). We observed a mean variation of FEV_1_ of +160 mL and FVC of +110 mL, but no significant difference was observed after HFNC therapy on FEV_1_ (f-ratio 0.79. *p*-value 0.45) and FVC (f-ratio 0.411. *p*-value 0.66) (Figure 4 and Figure 5).

**Figure 6:**
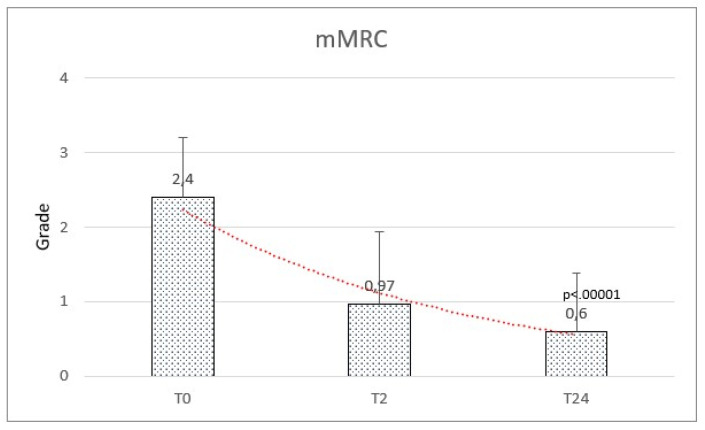
Modified Medical Research Council (mMRC) scale significantly improved from baseline (T0) to follow-up after 2 months (T2) and after 2 years (T24).

The mMRC score at baseline ranged between 0 and 4, with a mean of 2.40 (±0.81). A significant reduction in dyspnea was observed after HFNC therapy with a mean of 0.97 (±0.97) at 2 months and 0.60 (±0.78) at 24 months (f-ratio value 95.512. *p*-value < 0.00001. T1:T2 Q = 14.68 (*p* = 0.000001). T1:T3 Q = 18.52 (*p* = 0.000001). T2:T3 Q = 3.84 (*p* = 0.019)) (Figure 6). The results are reported in Table 2.

**Figure 7:**
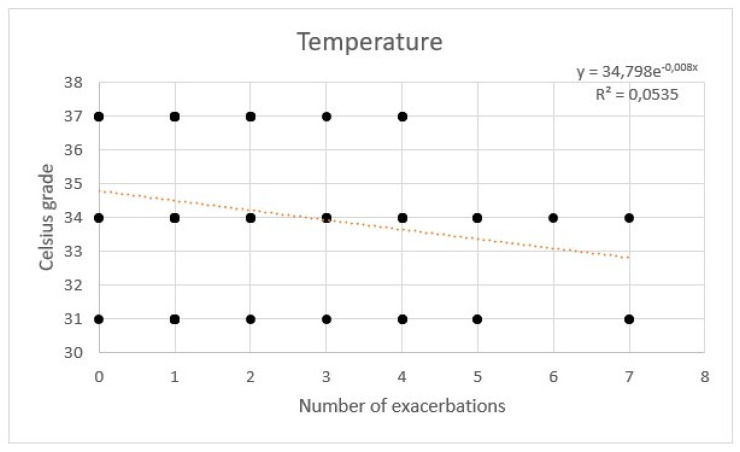
Regression of exacerbations rate and temperature of high flow nasal cannula.

**Figure 8:**
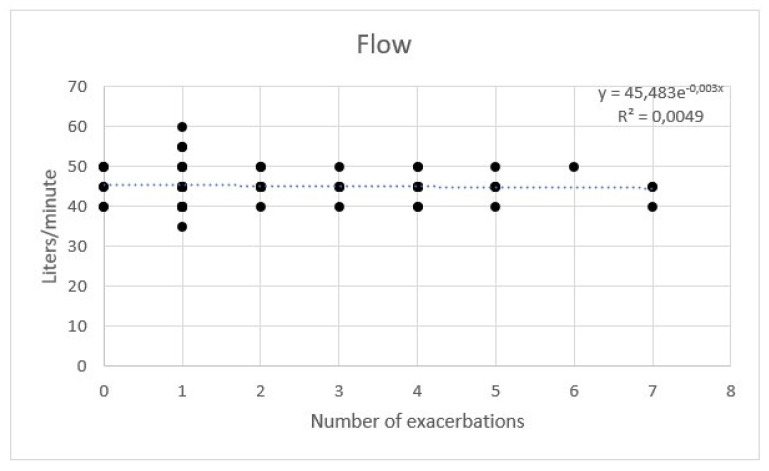
Regression of exacerbations rate and flow of high flow nasal cannula.

**Figure 9:**
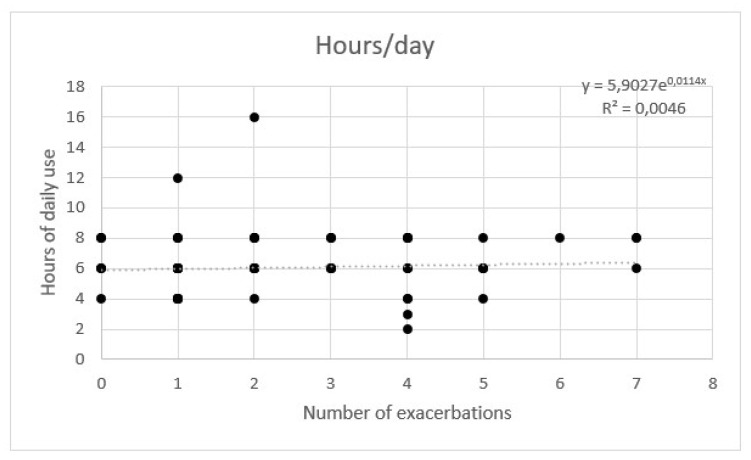
Regression of exacerbations rate and hours per day of high flow nasal cannula.

AE rate showed a weak correlation with temperature. No significant correlation between flow and hours per day was observed with a multivariate regression (Figure 7, Figure 8 and Figure 9).

## 5. Discussion

This single-center protocol depicts our experience with HFNC in bronchiectasis. HFNC is conceived as an add-on therapy and is performed as a part of a rehabilitation plan. There is limited evidence on long-term HFNC for chronic respiratory diseases, especially bronchiectasis. In a 6 weeks crossover study, 10 patients affected by interstitial lung disease (ILD) and chronic respiratory failure were treated with domiciliary HFNC; authors observed no differences in lung function and blood gasses, but walking distance at 6-min walking test (6 MWT) improved significantly [13]. Similarly, Chihara et al. reported an improvement in walking distance following four weeks of exercise training using an HFNC; in this study, HFNC was set with a flow of 50 L/min, and FiO2 was 1.0, involving chronic respiratory failure [14]. Our patients were not hypoxemic, and no additional oxygen was used with HFNC; nevertheless, we observed a significant improvement in dyspnea and functional limitation scored by the mMRC scale in the absence of a significant modification of lung function.

This result substantially remarks that HFNC achieves therapeutic effects besides conventional oxygen therapy (COT). Bronchiectasis is characterized by increased mucus production and expectoration. The standard of care for bronchiectasis should include mucus normalization and reduction of sputum, but also the regularity and ease of drainage of deep bronchial secretions. Humidification has shown some benefit in bronchiectatic patients; in fact, domiciliary heated and humidified air therapy for 7 days improves lung mucociliary clearance [6]. On the contrary, an in vitro test on the ovine tracheal model confirmed that a reduction in air temperature with 100% relative humidity changes mucus transport velocity and ciliary beat frequency, resulting in epithelial damage [15].

The physiological effects of HFNC on persistent airway inflammation with mucus retention in patients with chronic airway disorders such as COPD or bronchiectasis have been described by Rea et al. [16]. In this randomized controlled trial involving 108 patients, 1-year humidification therapy significantly reduced exacerbation days and improved lung function and quality of life. In this study, subjects were prevalently COPD and showed a more compromised lung function at baseline compared to our study. This can explain the relevant results, especially on spirometric parameters. Most likely, there are many mechanisms behind HFNC therapy efficacy. The prolonged use of heated and humidified airflow may reduce respiratory tract infections by improving airway inflammation and mucociliary clearance. In parallel, applying a high flow of air and a minimum positive end expiratory pressure (PEEP) result in the enhancement of small airways function, gas exchange, and dead space washout [17]. However, despite current literature, the underlying mechanism by which HFNC can improve outcomes remains speculative.

The major aim in chronic respiratory diseases is the reduction of exacerbations. AEs are a common cause of reduced quality of life (QoL), uncontrolled symptoms, and poor prognosis. At the same time, AE is associated with emergency room access, hospitalization, and loss of autonomy and is principally responsible for the economic burden of the disease. For this reason, the first outcome of our study was the reduction of AEs per year, and the follow-up was set at 2 years. A randomized controlled trial was conducted on 200 COPD patients with chronic hypoxemic respiratory failure treated with long-term oxygen therapy. They were randomized to receive HFNC as add-on therapy. The average daily use of HFNC was 6 h/day. The HFNC group had a lower AE-COPD rate; no difference was observed in all-cause mortality, but the follow-up was at 12 months [18]. Our paper highlights that HFNC reduces AE in all-causes adult bronchiectasis, even in the absence of hypoxemia.

Few studies are available for long-term HFT in adult patients with a primary diagnosis of bronchiectasis and frequent exacerbations. In addition, comparable results on exacerbations have been observed with different settings of temperature and flow. Essentially, the patient’s comfort should be taken into account. HFNC is generally a very well-tolerated therapy. This is a key feature to guarantee long-term home therapy. We observed prolonged compliance of 95% during the observation period. The compliance was estimated as the use of HFNC for at least 2 h per day and at least 4 days per week. This device showed a low incidence of adverse events. In our study, we observed 1 case of minor epistaxis, 1 case of interface discomfort, and 2 cases of dysphagia.

Potential risks have been reported in the literature. Respiratory tract infections, bronchospasm, dyspnea, onset or worsening of pneumothorax and pneumomediastinum, interface discomfort, nasal dryness, oral dryness, eye irritation, and nasal or eye trauma are to be considered very rare. Hemoptysis and nasopharyngeal bleeding are rare. Dysphagia, abnormalities of swallowing, altered bolus progression, and gastric distention are not frequently reported but have not been widely investigated. The same adverse effects are acknowledged and definitely more common with other respiratory therapies, such as oronasal dryness and bleeding, during conventional oxygen therapy (COT). Not considering the comparison with continuous positive airway pressure (CPAP) or non-invasive ventilation (NIV). CPAP is complicated by interface discomfort and skin lesions in virtually one hundred percent of patients when performed in chronic care settings.

As a matter of fact, there is no direct comparison between heated and humidified nasal cannula and CPAP in bronchiectasis patients when it comes to long-term home care. The main reason is the absolute lack of recent studies, even though the non-invasive clearance of airway secretions has been widely used throughout the decades. Airway clearance techniques are potentially beneficial for various diseases that have known clearance abnormalities [19]. In 1996, Hardy affirmed that patients with diseases known to cause clearance abnormalities could improve their sputum clearance with some techniques, such as positive expiratory pressure, autogenic drainage, and active cycle of breathing techniques. Previous studies demonstrated that the use of the forced exhalatory technique in patients with nonproductive cough still resulted in the movement of secretions proximally from all regions of the lung in patients with airway obstruction. It is, therefore, reasonable to consider airway clearance techniques for any patient who has a disease known to alter mucous clearance, including CF, dyskinetic cilia syndromes, and bronchiectasis from any cause. Patients with atelectasis from mucous plugs and hypersecretory states, such as asthma and chronic bronchitis, patients with pain secondary to surgical procedures, and patients with neuromuscular disease, weak cough, and abnormal patency of the airway may also benefit from the application of airway clearance techniques. CPAP alone has been shown to improve achievable flow rates that will increase air–liquid interactions for patients with these diseases or airway malacia. The use of positive pressure to maintain airway patency in these children allows cephalad clearance of secretions. Patients with segmental atelectasis, particularly related to asthma, may benefit from an intrapulmonary percussive ventilator, positive expiratory pressure, or PDPV. Prevention of postoperative atelectasis is particularly well suited to positive expiratory pressure, which is not as painful as techniques using oscillations. Optimizing ventilation in patients with such abnormalities may require positive pressure ventilation either during sleep or continuously.

Since then, expert opinion has led to CPAP or NIV in the acute setting. It is more advisable in the presence of respiratory failure with hypercapnia or acidosis. Ventilatory pump failure is also an indication of respiratory support. Bronchiectasis and atelectasis secondary to trauma or surgery can be effectively prevented by HFT. However, concomitant conditions such as obesity, hypoventilation, and prolonged immobilization can require CPAP/NIV.

## 6. Limitations

This study has several limitations. A single center was involved, with a small sample size. This reflects a generalized difficulty in diagnosing bronchiectasis. The incidence of the disease is often underestimated, especially when a history of smoking leads to a subsequent chronic obstruction. Similarly, the diagnosis of bronchiectasis is confounded in primary forms when etiology is attributed to the recurrent infective episodes in the first decades of life.

Despite the limited number of patients, we tried to consider the research priorities for bronchiectasis, and for this reason, the focus was on exacerbations, which are responsible for the major burden of the disease. AEs characterize the natural evolution of the disease but also, are independently related to the anatomical extension of lung damage and lung function decline over the years. AEs are very likely to condition morbidity and mortality of bronchiectasis.

Another limit is the number of questionnaires administered during the study. Dyspnea was prioritized among symptoms because it has a major impact on the patient’s performance status compared to cough and sputum. In fact, the mMRC scale evaluates the role of dyspnea during daily routine and reflects physical limitations.

Finally, no definitive recommendations can be made about a single approach, but a dynamic evaluation of the single case is to be endorsed. Further multicentric randomized trials are needed.

## 7. Conclusions

There is very little evidence investigating the effects of HFNC in bronchiectasis. Adult patients can develop a severe clinical status characterized by persistent infection, inflammation, and progressive tissue destruction. Anatomical changes of the bronchi wall and mucoid impaction are frequently observed in severe bronchiectatic syndrome. According to our experience, HFNC improves alveolar ventilation and epithelial clearance, thus preventing mucus retention and atelectasis. We observed that long-term home HFT is well tolerated in chronic stable adult bronchiectasis. In the long run, HFNC reduced the rate of exacerbations and the rate of hospitalization. The mMRC dyspnea grade improved rapidly after HFT initiation, and this result was confirmed at the final 2-year follow-up. No significant effect on lung function was observed. In conclusion, HFT is a valid approach to implement for rehabilitation in all-causes adult bronchiectasis.

## Figures and Tables

**Table 1:** Baseline features of study population.

Patients.	78	
Females (%)	42	53.8%
Median age (IQR)	70	60–76
Etiology:		
Respiratory tract infection/pneumonia	40	51.3%
COPD	20	25.7%
Congenital	9	11.5%
Idiopathic	5	6.4%
ILD	1	1.3%
Surgery/Trauma	3	3.8%
Concomitant medications:		
No	29	37.2%
LAMA	10	12.8%
ICS/LABA	17	21.8%
LABA/LAMA	6	7.7%

COPD: chronic obstructive pulmonary disease. ILD: interstitial lung disease. LAMA: long-acting muscarinic antagonist. ICS: inhaled corticosteroids. LABA: long-acting β2 agonists.

**Table 2:** Outcomes of long-term high flow nasal cannula therapy.

Outcomes	T0	T1	T24	One-Way ANOVA Test
Exacerbations				
Mean (SD)/year	2.81 (±2.15)	2.36 (±0.69)	0.45 (±0.66)	F = 79.703; *p* < 0.00001
0–1	26 (33.3%)	70	69 (88.5%)	
≥2	52 (66.7%)	8	9 (11.5%)	
Hospitalization	45 (57.7%)	22 (25.2%)	8 (10.2%)	
Mean (SD)/year	1.65 (±2.10)	1.25 (±1.68)	0.56 (±0.98)	F = 76.165; *p* < 0.00001
Spirometry:				
FEV_1_ [Mean (SD)] mL	2.39 (±0.87)	2.54 (±0.85)	2.55 (±0.82)	F = 0.79; *p* = 0.454
FVC [Mean (SD)] mL	2.73 (±0.88)	2.84 (±0.91)	2.84 (±0.90)	F = 0.41; *p* = 0.664
Compliance	78 (100%)	78 (100%)	74 (95%)	
mMRC				
Mean (SD)	2.40 (±0.81)	0.97 (±0.97)	0.60 (±0.78)	F = 95.512; *p* < 0.00001
0–1	51 (65.4%)		66 (84.6%)	
2–5	27 (34.6%)		12 (15.4%)	

SD: standard deviation. FEV1: forced expiratory volume in the first second. FVC: forced vital capacity. mMRC: modified medical research council.

## Data Availability

Collected data are available at the A.O. dei Colli records archive.
